# Living With Bipolar Disorder and Medication PRN—A Case‐Report

**DOI:** 10.1111/bdi.70086

**Published:** 2026-02-04

**Authors:** Annemiek Dols, John Brennikmeijer, Jim van Os

**Affiliations:** ^1^ Department of Psychiatry, UMC Utrecht Brain Center University Medical Center Utrecht Utrecht the Netherlands; ^2^ ValueMachines BV Losser the Netherlands

## 1 Case Presentation

**FIGURE 1:**
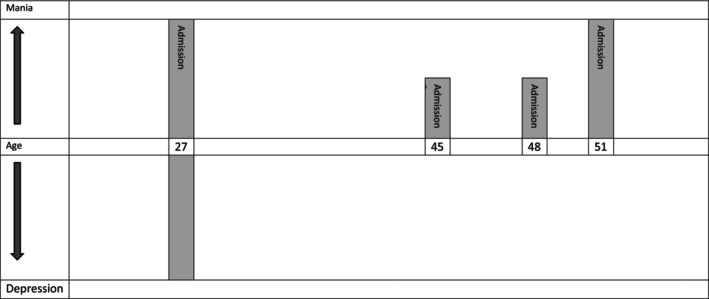
Life‐chart of our case.

John is a 54‐year‐old businessman diagnosed with bipolar disorder (BD) type I after experiencing his first psychotic manic episode at the age of 27, which required hospitalization. He was discharged with valproic acid and clonazepam, which were gradually tapered over the following year by his psychiatrist. At age 39, he experienced another psychotic manic episode, which was stabilized with valproic acid and benzodiazepines, though hospitalization was not required. At age 47, he voluntarily admitted himself for 1 day due to a manic episode and was prescribed olanzapine at 20 mg per day. At age 51, John was arrested multiple times during COVID‐19 demonstrations Figure 1. Additionally, his colleagues at his company were worried about their reputation, and he refused to stop the weekly protests on Sunday, leading to an involuntary admission on a Saturday afternoon the day before a planned protest. However, the judge did not uphold the involuntary admission, and John was discharged at his request after 3 days, having taken olanzapine 5 mg once to manage his mania.

There is a family history for mood disorders; his aunt on his mother's side has been diagnosed with BD and died by suicide. Additionally, his father's brother has been diagnosed with either BD or psychosis and spent decades in a hospital. John consumes 20–30 units of alcohol per week and is in good physical health.

Throughout his life, John has had several periods of outpatient care during which his medication was tapered upon his request. He reports experiencing hypomanic episodes 2–3 times per year and depressive episodes every 2–3 years. He follows a PRN (pro re nata, or “as needed”) regimen for olanzapine and keeps daily records of sleep duration, physical exercise, weight, alcohol intake, life‐events, and mood on a life‐chart.

John has also trained himself in self‐management through a workshop by Tom Wootton, an expert by experience and author of the book *Bipolar IN Order*. For instance, if he feels manic and is inclined to speak with everyone he encounters, he has learned to control himself and avoid acting on that impulse. He also uses practical techniques, such as an hourglass, to wait for others to finish speaking during webinars. John has established life rules, such as not working on Sundays or outside the hours of 7:00 a.m. to 10:00 p.m.

The *Bipolar IN Order* approach has been beneficial for John as it focuses on learning to function effectively during mood states rather than solely aiming for symptom reduction or complete remission. This approach recognizes that complete remission may not be attainable and instead emphasizes extending stability during both manic and depressive episodes. It incorporates a unique perspective on time, encouraging individuals to recognize early signs of mood changes and develop proactive strategies to maintain stability, regardless of their emotional state. To extend his comfort zone gradually, John initially practiced maintaining control for 1 day, then for several days, and eventually for a week. By “accounting for time” he designates in advance how much time he will allow himself to feel manic (and thus be outside his comfort zone) before intervening with behavioral adjustments or PRN medication. This approach has empowered him to use personalized tools and techniques suited to his mood state, fostering confidence in his ability to manage his mental health by recognizing the role of time in his functioning.

John is also a member of the *Bipolar Social Club* set up by entrepreneur Paul English who argues that stigma and secrecy are the enemies of healing.

## 2 Discussion

John's experience highlights the importance of integrating knowledge gained through lived experience into psychiatric practice. This is not merely an optional addition; it may be essential for creating a person‐centered approach. Frameworks like Tom Wootton's *Bipolar IN Order* demonstrate the value of shifting the focus from symptom reduction to creating a meaningful coexistence with mood swings, even learning to harness their strengths. This requires a commitment to epistemic pluralism—a respect for multiple ways of “knowing”—where clinical expertise and experiential knowledge are seen not as competing but as complementary. For psychiatrists, developing conceptual competence becomes crucial, allowing them to better understand and engage with diverse perspectives. It is in this intersection of clinical and experiential understanding that meaningful progress happens, helping us to support individuals like John in ways that resonate with their lived reality.

Despite minimal financial barriers to accessing mental health care in the Netherlands, only 51% of individuals meeting the DSM‐5 lifetime criteria for BD had contact with mental health services in the past year, and just 43% used medication during that period, according to recent data from the Netherlands Mental Health Survey and Incidence Study (NEMESIS (trimbos.nl)). This real‐world scenario contrasts sharply with the recommendations in most national and international guidelines, which advocate for the long‐term use of prophylactic medication even after a first episode. However, this finding aligns with estimated rates of nonadherence in BD, as reported by Chakrabarti [1], which are comparable to nonadherence rates in other chronic illnesses. In BD, demographic and illness‐related factors have not consistently explained or predicted nonadherence, suggesting other influential factors. These may include stigma related to BD and its treatment, the quality of the therapeutic relationship with the prescriber, personal perceptions of mood instability and its causes, beliefs and attitudes toward medication, levels of social support, and of course, access to mental health care and medication.

Adherence is defined as “the extent to which taking medication corresponds with agreed recommendations from a health care provider.” Nonadherence is often viewed as an undesirable situation that negatively impacts patient outcomes. However, can it also be seen as an opportunity to learn from patients? With approximately half of individuals with BD not taking medication, an important question arises: how do they manage their mood fluctuations?

It is well‐known that maintenance medication does not guarantee the prevention of new episodes. Studies report a relapse risk of 31.4% for those on active treatment versus 51.1% for those on placebo [2], indicating that nonadherent individuals still have nearly a 50% chance of avoiding relapse during follow‐up periods ranging from 24 to 104 weeks. A naturalistic study examining the long‐term effects of continuing versus discontinuing maintenance treatment found that, at a 6‐year follow‐up, recurrence rates were significantly higher in patients who discontinued lithium (80%) compared to those who continued treatment (40%) [3]. In the discontinuation group, recurrence primarily occurred within the first 2 years, while in the maintenance group, recurrence happened gradually over the 6‐year period. Furthermore, a review found that discontinuing maintenance medication for BD for more than a month was associated with an increased risk of relapse; nevertheless, 47.3% of patients who stopped their medication for longer than 6 months did not experience a relapse [4]. These findings highlight the complexity of medication adherence in BD and suggest a potential value in understanding the strategies that nonadherent patients use to manage their symptoms.

Many individuals experience side effects from mood stabilizers and antipsychotics prescribed as prophylactic treatment for BD. These include immediate, troublesome effects such as tremors, concentration difficulties, emotional blunting, and cognitive slowing, as well as serious long‐term health risks, including metabolic syndrome, which heightens the risk of cardiovascular diseases, thyroid disorders, and renal failure. Furthermore, many individuals eventually express a desire to live without medication, even if they are not experiencing significant side effects.

Psychological therapies for individuals with BD are often neither systematically considered nor routinely offered as a stand‐alone treatment option [5]. When such therapies are provided, they are almost always adjunct to mood‐stabilizing medication. Common psychological intervention packages, such as psychoeducation, family‐focused therapy, cognitive behavioral therapy, interpersonal and social rhythm therapy, dialectical behavior therapy, and acceptance and commitment therapy, share core elements like psychoeducation, self‐management, and emotional regulation. However, their frequent emphasis—whether implicit or explicit—on medication adherence and symptom reduction may not fully support personal recovery goals.

This raises a critical question: should psychoeducation and other psychological therapies replace medication as the cornerstone of treatment in BD? Considering the personal and long‐term health implications of lifelong medication, it may be worth exploring whether a more individualized approach, one that centers on self‐management as a primary treatment, could better support sustainable recovery and quality of life.

## 3 Future Directions

Now it is the time to study the extent to which self‐management, lifestyle adjustments, stress reduction (through training in effective coping strategies), and trauma‐focused treatments can contribute to maintaining stability. It is crucial to explore when and for whom maintenance medication might be reduced or even discontinued. A well‐defined emergency plan for recognizing early signs of relapse along with easy access to (clinical) support is essential. PNR (as needed) medication may also be part of this plan. As clinicians, we recognize that we are only seeing part of the broader picture—those with BD who attend our mental health facilities. The other 50% who do not seek regular clinical care represent a valuable source of insight, potentially informing strategies that could enhance our approach to care.

## Funding

The authors have nothing to report.

## Ethics Statement

The publication was cocreated with the patient.

## Conflicts of Interest

The authors declare no conflicts of interest.

## Data Availability

Data are available upon resonable request.

## References

[1] S. Chakrabarti , “Medication Non‐Adherence in Bipolar Disorder: Review of Rates, Demographic and Clinical Predictors,” World Journal of Meta‐Analysis 5 (2017): 103, 10.13105/wjma.v5.i4.103.

[2] A. Nestsiarovich , C. E. S. Gaudiot , R. J. Baldessarini , E. Vieta , Y. Zhu , and M. Tohen , “Preventing New Episodes of Bipolar Disorder in Adults: Systematic Review and Meta‐Analysis of Randomized Controlled Trials,” European Neuropsychopharmacology 54 (2022): 75–89, 10.1016/j.euroneuro.2021.08.264. 34489127

[3] M. G. Biel , E. Peselow , L. Mulcare , B. G. Case , and R. Fieve , “Continuation Versus Discontinuation of Lithium in Recurrent Bipolar Illness: A Naturalistic Study,” Bipolar Disorders 9, no. 5 (2007): 435–442, 10.1111/j.1399-5618.2007.00389.x. 17680913

[4] T. Kishi , Y. Matsuda , K. Sakuma , M. Okuya , K. Mishima , and N. Iwata , “Recurrence Rates in Stable Bipolar Disorder Patients After Drug Discontinuation v. Drug Maintenance: A Systematic Review and Meta‐Analysis,” Psychological Medicine 51, no. 15 (2021): 2721–2729, 10.1017/S0033291720003505. 33046156

[5] K. Wright , M. Koenders , K. M. Douglas , et al., “Psychological Therapies for People With Bipolar Disorder: Where Are We Now, and What Is Next? ISBD Psychological Interventions Taskforce‐Position Paper,” Bipolar Disorders 26 (2024): 523–528, 10.1111/bdi.13418. 38632696

